# Retraction Note: Stress urinary incontinence: long-term results of laparoscopic Burch colposuspension

**DOI:** 10.1186/s12893-016-0141-6

**Published:** 2016-04-28

**Authors:** Domenico Prezioso, Fabrizio Iacono, Giovanni Di Lauro, Ester Illiano, Giuseppe Romeo, Antonio Ruffo, Nicola Russo, Bruno Amato

**Affiliations:** Department of Urology, University Federico II of Naples, Via S. Pansini, 5, Naples, 80131 ITALY; Hospital Santa Maria delle Grazie, Pozzuoli, Naples Via Domiziana, Loc. La Schiana, Pozzuoli, Naples Italy; Department of General, Geriatric, Oncologic Surgery and Advanced Technologies, University “Federico II” of Naples, Via Pansini, 5, Naples, 80131 Italy

## Retraction Notice

This article [1] has been retracted by the authors because it contains large portions of text that were duplicated from a number of pervious publications including Rofeim et al. [2], Carey et al. [3] and Hong et al. [4]. The authors apologise for failing to cite these articles.

## Notes

The online version of the original article can be found at 10.1186/1471-2482-13-S2-S38.
